# SNPs of ACE1 (rs4343) and ACE2 (rs2285666) genes are linked to SARS-CoV-2 infection but not with the severity of disease

**DOI:** 10.1186/s12985-022-01782-6

**Published:** 2022-03-19

**Authors:** Nahid Alimoradi, Moein Sharqi, Dena Firouzabadi, Mohammad Moein Sadeghi, Mohammad Iman Moezzi, Negar Firouzabadi

**Affiliations:** 1 Department of Pharmacology & Toxicology, School of Pharmacy, Shiraz University of Medical Sciences, Shiraz, Iran; 2 Student Research Committee, Shiraz University of Medical Sciences, Shiraz, Iran; 3 Shahid Faghihi Hospital, Shiraz University of Medical Sciences, Shiraz, Iran; 4 Clinical Pharmacy Department, School of Pharmacy, Shiraz University of Medical Sciences, Shiraz, Iran

**Keywords:** COVID-19, Renin-angiotensin system, Angiotensin-converting enzyme, ACE polymorphism, Genetic association, rs2285666, rs4343

## Abstract

COVID-19 and the renin-angiotensin system (RAS) are linked by angiotensin-converting enzyme 2 (ACE2), a key enzyme in RAS that has been validated as a SARS-CoV-2 receptor. Functional ACE1/ACE2 gene polymorphisms may lead to the imbalance between ACE/ACE2 ratio and thus generating RAS imbalance that is associated with higher degrees of lung damage in ARDS that may contribute to the COVID-19 infection outcome. Herein, we investigated the role of RAS gene polymorphisms, ACE1 (A2350G) and ACE2 (G8790A) as risk predictors for susceptibility and severity of COVID-19 infection. A total of 129 included: negative controls without a history of COVID-19 infection (n = 50), positive controls with a history of COVID-19 infection who were not hospitalized (n = 35), and patients with severe COVID-19 infection who were hospitalized in the intensive care unit (n = 44). rs4343 of ACE and rs2285666 of ACE2 were genotyped using PCR–RFLP method. Our results indicated that susceptibility to COVID-19 infection was associated with age, GG genotype of A2350G (Pa = 0.01; OR 4.7; 95% CI 1.4–15.1 and Pc = 0.040; OR 2.5; 95% CI 1.05–6.3) and GG genotype of G8790A (Pa = 0.044; OR 6.17; 95% CI 1.05–35.71 and Pc = 0.0001; OR 5.5; 95% CI 2.4–12.4). The G allele of A2350G (Pa = 0.21; OR 1.74; 95% CI 0.73–4.17 and Pc = 0.007; OR 2.1; 95% CI 1.2–3.5) and G allele of G8790A (Pa = 0.002; OR 4.26; 95% CI 1.7–10.65 and Pc = 0.0001; OR 4.7; 95% CI 2.4–9.2) were more frequent in ICU-admitted patients and positive control group. Also lung involvement due to COVID-19 infection was associated with age and the comorbidities such as diabetes. In conclusion, our findings support the association between the wild genotype (GG) of ACE2 and homozygote genotype (GG) of ACE1 and sensitivity to COVID-19 infection, but not its severity. However, confirmation of this hypothesis requires further studies with more participants.

## Introduction

Severe acute respiratory syndrome coronavirus 2 (SARS-CoV-2) emerged in the Wuhan province of China in late 2019 and had soon spread vastly worldwide. On March 11^th^ 2020, the coronavirus disease-19 (COVID-19) was announced as a global pandemic by the WHO. The highly contagious and pathogenic disease has led to about 4.5 million deaths worldwide ever since [1, 2]. Surprisingly the death rate is not evenly distributed throughout the world and variously affects ethnicities. SARS-CoV-2 is a single-stranded RNA beta-coronavirus with a spike protein that can enter cells by binding to angiotensin-converting enzyme 2 (ACE2) as an approved receptor [3–5]. The spike (S) consists of a large ectodomain that includes a receptor-binding subunit S1 and a membrane fusion subunit S2. The S1 subunit has a receptor-binding domain (RBD) that recognizes ACE2. Moreover, virus/receptor binding is a vital initial step in viral infection [5–7]. The renin-angiotensin system (RAS) which has substantial role in many illnesses [8–10] is among the candidate targets both in the pathogenesis and in treatment of COVID-19 [11]. RAS is best known for its play in regulating blood pressure and electrolyte balance, thereby controlling cardiovascular and renal function [12]. Results of meta-analyses are indicative of increased mortality risk in co-existence of cardiovascular diseases and COVID-19 infection [13–15]. Clinical cohort studies advocate the possible association of unbalanced RAS with lung fibrosis and acute respiratory distress syndrome (ARDS) [16, 17] seen in COVID-19 patients. Alongside, angiotensin-converting enzyme inhibitors (ACEIs) and angiotensin receptor blockers (ARBs) has been shown to have protective effects in patients with COVID-19 by establishing this balance [18–20].

RAS and COVID-19 are linked by ACE2 that SARS CoV-2 uses as the functional receptor for cell fusion and induction of infections in the respiratory system [21–23]. ACE2 is a key enzyme in RAS and is found on the surface of lung alveolar epithelial cells, facilitating the entry of the SARS-CoV-2 [24]. ACE2 neutralizes the effects of Angiotensin II (Ang II) by turning it to the vasodilator peptide Ang (1–7). Ang II is a potent vasoconstrictor peptide in RAS and the main product of the enzyme ACE-1, converting Ang-I to Ang-II. On the other hand, ACE2 converts the ACE substrate, Ang-I, to Angiotensin (1–9). ACE2 exerts opposite effects on ACE action by two different mechanisms [25–27].

The protective effects of ACE2 have been observed in various experimental models of acute lung failure that may contribute to COVID-19 treatment (7, 8). The vital role of ACE2 in COVID-19-induced lung injury has been repeatedly demonstrated [28]. COVID-19-induced inflammation begins with the binding of ectodomain S1 of SARS-CoV-2 to ACE2. After membrane fusion and decline in ACE2 levels, metabolism of Ang II disrupts [29]. Elevated levels of Ang II stimulates the release of inflammatory cytokines and leads to local inflammation [30]. Pulmonary vascular inflammation leads to ACE1 shedding phenomenon and an increase in its releases into the interstitium, which, in turn, exacerbates incline in Ang II generation and leukocyte infiltration [28, 31]. Following Ang II/ATR1 over-interactions, ROS production increases and as a result aggravates systemic inflammation in COVID-19 infection by increasing the production of inflammatory factors like tumor necrosis factor-alpha (TNF-a), Interleukin-6 (IL-6), and C-reactive protein (CRP) [32–34].

ACE2 is a polymorphic gene with about 140 single nucleotide polymorphism (SNP) loci determined on the human genome [35]. Many studies have identified various SNPs on ACE2 that may be involved in COVID-19 [36–38]. But only a handful of these options have been clinically tested; examples of these variants that have been recently studied are rs2106809 and rs2285666 [39, 40]. Among the functional SNPs identified on the ACE2 gene, G8790A (rs2285666) located on chromosome Xp22 in intron 3 suggests that this variant may alter mRNA splicing and thus affect ACE2 gene expression [41]. Some genetic variants in the ACE2 can bring about variations in binding affinity of ACE-2 for SARS COV-2 RBD [42, 43]. rs2285666 is one of these SNPs whose wild type enhances ACE2 production with a greater affinity for SARS-CoV-2 [44].

The other SNP studied in the present study, is A2350G (rs4343), a functional variant located on exon 17 of ACE1 gene. Considering the effects of this polymorphism on the activity and serum level of the ACE-1 enzyme [45, 46], it might be postulated that carriers of specific genotypes of this variant may be more susceptible to COVID-19 [45, 47].

In the present study we hypothesized the association between rs4343 and rs2285666 with susceptibility and severity of COVID-19. To the best of our knowledge, the link between ACE2 gene (G8790A) variants and COVID-19 has not been studied yet in the Iranian population and the association between ACE1 gene (A2350G) and COVID-19 has not been studied in any ethnicity so far.

## Materials and methods

### Ethics statement and patients collection

This study was approved by the Research Ethics Committee of Shiraz University of Medical Sciences with the ethical code of IR.SUMS.REC.1399.293 and conducted under the ethical principles of the World Medical Association (Helsinki Declaration). The study population in this case–control study comprises 129 cases which were classified into three groups: healthy controls with no history of COVID-19 infection to date, patients with a history of COVID-19 infection who were not hospitalized and patients who suffered severe COVID-19 and were hospitalized in the intensive care unit (ICU) of Shiraz Shahid Faghihi Hospital, the main referral center for management of COVID-19 in Shiraz, Iran (Table 1).

**Table 1:** Demographic properties and co-morbidities of enrolled subjects and their relation with the possibility to COVID-19 infection

Variables	Negative (N = 50)	Positive (N = 35)	ICU (N = 44)	Total (N = 129)	Pa (< 0.05)
Sex, n (%)					0.991
Female	24 (48)	20 (57.15)	18 (41)	62 (48.1)	
Male	26 (52)	15 (42.85)	26 (59)	67 (51.9)	
Age (years)	37.5 ± 14.5	39.5 ± 14.8	56.5 ± 15.5		0.008
BMI (kg/m^2^)	23.7 ± 4.9	24.7 ± 3.6	23.9 ± 2.0		0.433
Occupation (Health care personnel/Others), n (%)	22/28 (44/56)	18/17 (51.4/48.6)	13/31 (29.5/70.5)		0.216
Cardiovascular dx, n (%)	3 (6)	1 (2.85)	8 (18.18)	12 (9.3)	0.577
Diabetes, n (%)	1 (2)	1 (2.85)	1 (2.27)	3 (2.3)	0.304
Immunodeficiency dx, n (%)	2 (4)	1 (2.85)	1 (2.27)	4 (3.1)	0.845

ICU, Intensive care unit; Pa, Adjusted *P* value; BMI, Body mass index

Inclusion criteria of COVID-19 patients were as follows:

Diagnosis of COVID-19 was made based on patients’ clinical status as defined by World Health Organization [2] and a positive PCR test [48]. The positive control group included individuals with a history of PCR confirmed Covid-19 infection with mild to moderate symptoms. According to WHO definition for mild disease, patients with mild pulmonary or extra pulmonary symptoms, showing no hypoxia, did not require further workup and hospital admission and were categorized as non-ICU admitted group of patients.

Individuals with no clinical confirmation of the infection accompanied by a negative PCR test, were considered as the healthy control group.

Demographic information of participants such as age, sex, and underlying medical conditions and past medication history was collected (Table 1). Extent of lung involvement was evaluated and categorized as minimal, intermediate and severe in each patient based on High Resolution Computed Topography (HRCT) results [49].

### DNA extraction and genotype determination

**Fig. 1:**
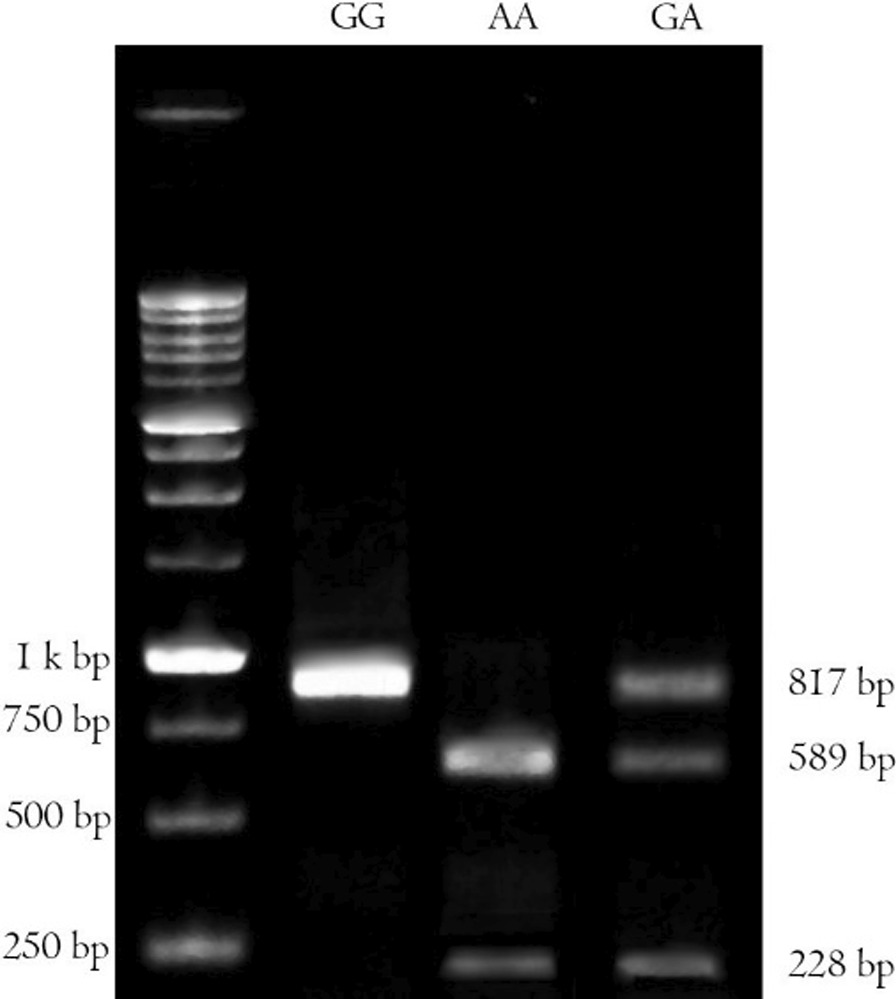
Agarose gel electrophoresis of the PCR–RFLP products of ACE2 G8790A digested with AluI restriction enzyme. The GG genotype was recognized as a single band at 817 bp, the AA genotype as two bands at 589 and 228 bp and the GA genotype as three bands at 817, 589 and 228 bp

**Fig. 2:**
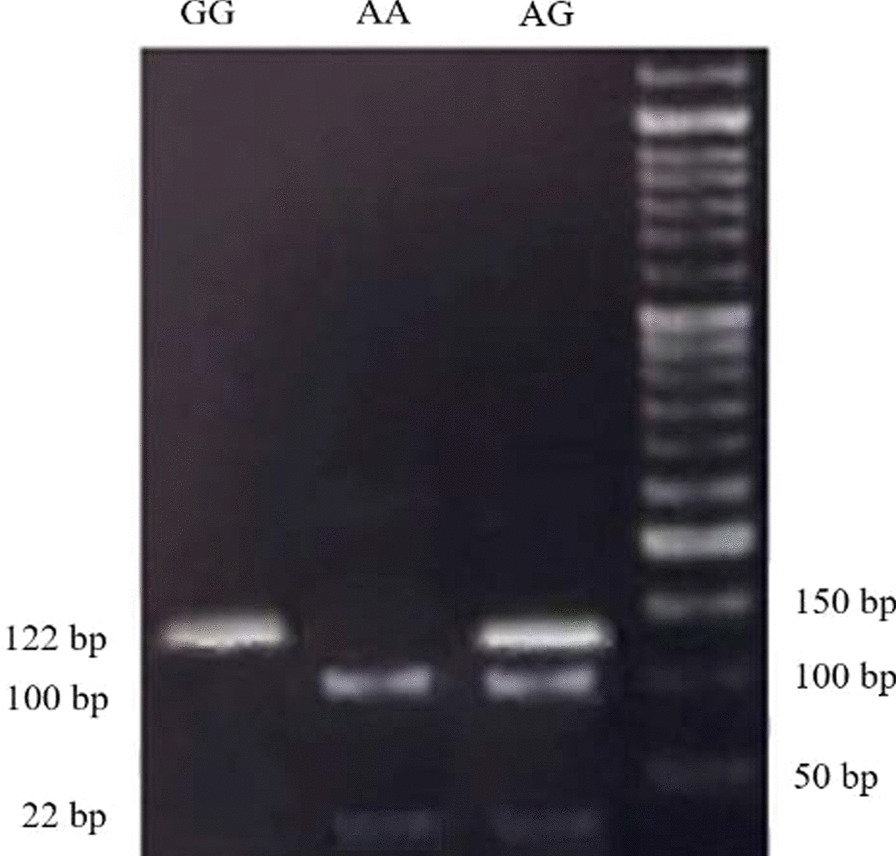
Agarose gel electrophoresis of the PCR–RFLP products digested with BstUI restriction enzyme. The GG genotype was recognized as a single band at 122 bp, the AA genotype as two-bands at 100 and 22 bp and the AG genotype as three-bands at 122, 100 and 22 bp

The blood samples were collected and DNAs were extracted from leukocytes of whole blood using a boiling method as described previously by Miller et al. [50]. DNA extraction efficiencies were assessed using NanoDrop®. The extracted DNAs were stored at -20 °C for polymerase chain reaction restriction fragment length polymorphism (PCR–RFLP) analysis. PCR amplification of G8790A and A2350G was performed using primers mentioned in Table 2 [51, 52]. PCR amplification/detection of G8790A was performed as described previously [53]. A total of 50 ng genomic DNA was mixed with 1 pmol of each PCR primer in a total volume of 25 µl containing 12.5 µl Master Mix (1X) (Ampliqon, Denmark). After PCR amplification at a primer annealing temperature of 60 °C, the products (10 µl) were digested with 1 U of AluI (Fermentas, Lithuania) at 37 °C for 16 h (Fig. 1). For A2350G genotyping, with a slight modification of a previously described protocol [45, 54], a total of 50 ng genomic DNA was mixed with 0.3 pmol of each PCR primer in a total volume of 20 µl containing 200 µM dNTPs, 2.5 mM MgCl2, and 0.3 mM of each primer and 1.25 U DNA Taq polymerase (Cinaclone, Iran). After initial denaturation at 96 °C for 5 min, PCR was carried out for 35 cycles, each one comprised of denaturation at 94 °C for 30 s, annealing at 60 °C for 30 s, and extension at 72 °C for 30 s, with a final extension time of 10 min at 72 °C. PCR products (7 µl) were digested with 0.5 U of BstUI (Fermentas, Lithuania) at 60 °C for 24 h. The digested products were run on a 3% agarose gel for 30 min at a 100 (v). G-allele was visualized as 122 bp and A-allele as 100-bp and 22-bp using a UV trans-illuminator (Fig. 2).

**Table 2:** List of forward and reverse primers for PCR–RFLP of rs4343 and rs2285666 and their associated restriction enzymes and DNA fragments

Polymorphism	Primer sequence (5′-3′)	TA (°C)	Restriction enzyme	DNA fragment size (bp)	References
A2350G (rs4343)	F-CTGACGAATGTGATGGCCGC R-TTGATGAGTTCCACGTATTTCG	61	BstUI 60 °C/24 h	122/100/22	[52]
G8790A (rs2285666)	F-TTCTCCCTGCTCCTATACTACCG R-TTCATTCATGTCCTTGCCCTTA	60	Alu1 37 °C/16 h	817/589/228	[53]

TA, the temperature of annealing; bp, Base pair

### Statistical analysis

Data were analyzed using SPSS® 23.0 for Windows (SPSS Inc., Chicago, Illinois) software. Data are demonstrated as mean ± SD for quantitative variables and percentages for categorical parameters. Chi-Square test (χ^2^) was used for comparing categorical parameters between groups. Hardy–Weinberg equilibrium (HWE) for the distributions of genotypes was estimated by chi-square (χ2) test. To estimate the association of genotypes and allele frequencies, and other variables with the possibility and severity of COVID-19 disease, we measured the odds ratio (OR) and the corresponding 95% confidence interval (CI) by multiple logistic regression analyses. All tests were two-sided and *P* < 0.05 was considered statistically significant**.**

## Results

Table 1 shows the demographic data of COVID-19 patients and healthy controls. Among 129 participants in our study, 51.9% were men and 48.1% were women, showing a female to male ratio of approximately 1:1. In the negative control group, the mean age and BMI were 37.5 ± 14.5 years and 23.7 ± 4.9 kg/m^2^ respectively. In the non-ICU admitted COVID-19 group mean age and BMI were 39.5 ± 14.8 years and 24.7 ± 3.6 kg/m^2^ respectively. Mean age and BMI of the ICU-admitted group were 56.5 ± 15.5 years and 23.9 ± 2.0 kg/m^2^ respectively. Regarding susceptibility to COVID-19 infection, sex and comorbidities showed no significant difference between controls vs COVID-19 patients neither using chi-square test nor after applying logistic regression (*P* > 0.05). However, severity of the disease was associated with diabetes (*P* = 0.033). Using logistic regression, there was significant difference regarding age between controls and COVID-19 patients (*P* = 0.008). Severity of the infection was associated with age in ICU-admitted patients and severe lung involvement was significantly more observed in older patients (*P* = 0.0001).

Lung involvement was diagnosed in 31.4% of the total study group. 61.4% and 22.7% of the ICU patients were diagnosed with severe and intermediate lung involvement based on HRCT records, respectively. Mortality rate was 21.7% (n = 28) among the patients. It is to note that all 28 patients were among the ones admitted to ICU.

Genotype and allele distribution of ACE1 and ACE2 gene polymorphism are presented in Tables 3 and 4. As shown, for ACE1 gene, the individuals with GG and GA genotypes were more susceptible to COVID-19 disease compared to the AA genotype (Pa = 0.01; OR 4.7; 95% CI 1.4–15.1 and Pc = 0.04; OR 2.5; 95% CI 1.05–6.3). The GG genotype of G8790A was associated with susceptibility to COVID-19 infection (*P* = 0.044; OR 6.17; 95% CI 1.05–35.71 and Pc = 0.0001; OR 5.5; 95% CI 2.4–12.4). The G allele of A2350G (Pa = 0.21; OR 1.74; 95% CI 0.73–4.17 and Pc = 0.007; OR 2.1; 95% CI 1.2–3.5) and G allele of G8790A (Pa = 0.002; OR 4.26; 95% CI 1.7–10.65 and Pc = 0.0001; OR 4.7; 95% CI 2.4–9.2) were more frequent in ICU and positive control groups. No significant association was observed in the severity of lung involvement due to COVID-19 disease and the outcome of the infection in the ICU-admitted group with different ACE 1 and 2 genotypes and sex (*P* > 0.05). Notably, patients with GG/GG genotypes (ACE1 and ACE2) were significantly more prone to COVID-19 infection (*P* = 0.008; OR 5.0, 95% CI 1.4–17.8).

**Table 3:** Genotype distribution in COVID-19 patients and healthy controls

SNP	Subjects (n)	Genotype frequencies (%)			Pa	OR; 95% CI	Pc	OR; 95% CI
		GG	GA	AA				
A2350G	Negative control (n = 50)	8 (16)	20 (40)	22 (44)	0.010	4.7; 1.4–15.1	0.040	2.6; 1.1–6.3
	Positive control (n = 35)	11 (31.5)	14 (40)	10 (28.5)				
	ICU-admitted patients (n = 44)	15 (34.1)	19 (43.2)	10 (22.7)				
G8790A	Negative control (n = 50)	24 (48)	19 (38)	7 (14)	0.044	6.2; 1.1–35.7	0.0001	5.5; 2.4–12.4
	Positive control (n = 35)	28 (80)	6 (17.2)	1 (2.8)				
	ICU-admitted patients (n = 44)	38 (86.3)	5 (11.4)	1 (2.3)				

Pa, adjusted P-value; Pc, P-value for Chi-Square test; OR, Odds ratio; CI, Confidence interval

**Table 4:** Allele frequencies in COVID-19 patients and healthy controls

SNP	Subjects (n)	Allele frequencies (%)		Pa	OR; 95% CI	Pc	OR; 95% CI
		A	G				
A2350G	Negative control (n = 50)	64 (64)	36 (36)	0.21	1.74; 0.73–4.17	0.007	2.1:1.2–3.5
	Positive control (n = 35)	34 (48.6)	36 (51.4)				
	ICU patients (n = 44)	39 (44.3)	49 (55.7)				
G8790A	Negative control (n = 50)	33 (33)	67 (67)	0.002	4.26; 1.7–10.65	0.0001	4.7; 2.4–9.2
	Positive control (n = 35)	8 (11.4)	62 (88.6)				
	ICU patients (n = 44)	7 (8)	81 (92)				

Pa: adjusted P-value; Pc: P-value for Chi-Square test; OR: Odds ratio; CI: Confidence interval

As shown in Table 5, subgroup analysis revealed that GG genotype of ACE2 was associated with COVID-19 both in female and male patients (*P* = 0.005, OR 5.2, 95% CI 1.7–16.5 and *P* = 0.002, OR 5.8, 95% CI 1.8–18.6, respectively). However, neither of the genotypes were associated with disease severity in neither sex (*P* > 0.05).

**Table 5:** Genotype distribution in COVID-19 patients and healthy controls disaggregated by sex

SNP	Sex	Subjects (n)	Genotype frequencies (%)			*P*	OR; 95% CI
			AA	GA	GG		
G8790A	Female	Non-COVID-19 (n = 24)	4 (17.7)	9 (37.5)	11 (45.8)	0.005	5.2; 1.7–16.5
		COVID-19 (n = 38)	2 (5.3)	5 (13.1)	31 (81.6)		
	Male	Non-COVID-19 (n = 26)	3 (11.5)	10 (38.5)	13 (50)	0.002	5.8; 1.8–18.6
		COVID-19 (n = 41)	0 (0)	6 (14.6)	35 (85.4)		
A2350G	Female	Non-COVID-19 (n = 24)	12 (50)	9 (37.5)	3 (12.5)	0.01	5.6; 1.4–22.3
		COVID-19 (n = 38)	8 (21)	13 (34.2)	17 (44.7)		
	Male	Non-COVID-19 (n = 26)	10 (38.5)	11 (42.3)	5 (19.2)	0.9	1.2; 0.35–4.0
		COVID-19 (n = 41)	12 (29.2)	20 (48.8)	9 (22)		

P, P-value; OR, odds ratio; CI, confidence interval

## Discussion

Results of our study indicated that the carriers of GG genotype of A2350G were significantly more prone to COVID-19. Regarding the ACE2 genetic variant, G8790A, our results advocate the association between GG genotype as well as its associated allele, the G allele, and the incidence of COVID-19.

ACE2, the entry receptor of SARS-CoV-2, is abundantly expressed in the respiratory and cardiovascular systems such as airway cells, alveolar epithelial type II cells, and endothelial cells [55–58]. Increased SARS-CoV-2/ACE2 binding, in addition to increased virus replication in target host cells, causes RAS imbalance [23, 41]. Binding of SARS-CoV-2 to ACE2 inhibits the high-affinity conversion of Ang II to Ang (1–7) by this enzyme [57, 59]. Past studies have shown that inhibition of ACE2, or ACE2 knockdown, significantly intensifies lung damage and the secretion of inflammatory cytokines [60]. An imbalance between ACE and ACE2 activity in favor of ACE activity is associated with generating RAS imbalance and higher degrees of lung damage in ARDS, which may be due to the reduction of pulmonary Ang-(1–7) levels and the elimination of its anti-inflammatory effects in the pulmonary system [61–64]. Increased AT1 receptor activity significantly worsens pulmonary function and edema that is associated with an increased in ACE activity and a decrease in ACE2 availability and the production of Ang-(1–7) [65, 66]. Ang- (1–7) regulates multiple intracellular signaling pathways and exhibits vasodilator, anti-proliferative, anti-inflammatory, and anti-fibrotic effects by binding to the Mas receptor [57, 67, 68].

G8790A (rs2285666) another SNP investigated in our study, is located in an intronic position that can alter mRNA splicing and affect gene expression and protein level of ACE2 [41, 69]. An investigation of the relationship between rs2285666 genotypes and circulating ACE2 in T2DM patients showed that the AA genotype has maximum expression level compared to other genotypes [70]. As we have observed in our study the wild genotype [71] and the G allele were significantly associated with the prevalence and risk of SARS-CoV-2 infection, similar to the results reported in the Indian and Caucasian populations [39, 72]. Moreover, in confirmation of previous studies, these variants did not affect the severity of the disease or the mortality rate of COVID-19 [73, 74]. SARS-CoV-2 induces ACE2 deficiency by down-regulation of ACE2, resulting in ACE1/ACE2 imbalance [75]. RAS imbalance at the level of the lung facilitates inflammatory and coagulation processes due to local Ang II overproduction and Ang-(1–7) deficiency [76, 77]. On the other hand, SARS-CoV-2 has an intrinsically high affinity for ACE2 receptors, and a mild or moderate ACE2 deficiency cannot play a protective role on host defense against viral invasion [78, 79]. As our results showed, age and the comorbidities such as diabetes that were previously reported to be associated with ACE2 deficiency can exacerbate COVID-19 induced-ACE2 deficiency and increase the severity and mortality rate of the disease [75].

Previous studies have shown that the G allele of ACE1 A2350G SNP in the ACE1 gene is associated with higher ACE activity and its serum concentrations. Hence, it can be concluded that in COVID-19 this variant may lead to increased levels of Ang II and subsequent inflammation [47, 80, 81]. Activation of AT receptors by Ang II, in addition to increasing vasoconstriction, leads to endothelial damage and endovascular thrombosis with activation of the coagulation cascade [82–84], which is observed in COVID-19 patients [85, 86]. ACE and ACE2 have divergent physiological functions. Because of the important role of RAS in the pathogenesis of cardiovascular, respiratory diseases and diabetes, cross-models of ACE and ACE2 genotypes may exacerbate COVID-19 by causing RAS imbalance through the increase in the increasing ACE/ACE2 ratio [87–91].

In our study, it was shown that gender was not significantly associated with the severity and incidence of COVID-19 disease, while previous studies have shown that men are more likely to develop severe COVID-19 disease., Also, similar to previous studies, our results showed the effect of age on the incidence and severity of COVID-19 disease [74, 92, 93].

As the study limitation, we should allude to the relatively small sample size of the enrolled subjects. However, the results of our study, especially regarding the ACE1 genetic variant, A2350G, which has not been studied in any other populations to date, may provide preliminary insights for further investigations in various ethnicities.

In conclusion, significant associations with COVID-19 susceptibility were identified for A2350G and G8790A polymorphism. In this study, we identified the possible risk genotypes, wild genotype (GG) of ACE2 and homozygote genotype (GG) of ACE1, for COVID-19 susceptibility. Meanwhile, neither of the variants of A2350G and G8790A were associated with the severity of COVID-19 in our study population. However, confirmation of this hypothesis requires further studies with more participants.

## Data Availability

The datasets used and/or analyzed during the current study are available from the corresponding author on reasonable request.

## References

[1] Yang, W., et al. (2020) *The role of imaging in 2019 novel coronavirus pneumonia (COVID-19).* European radiology 30: 1-9. 10.1007/s00330-020-06827-4 PMC7156903 32296940

[2] WHO. *Weekly operational update on COVID-19 - 16 August 2021*. 2021; Available from: https://www.who.int/publications/m/item/weekly-operational-update-on-covid-19. 16 August 2021

[3] Li F (2016). Structure, function, and evolution of coronavirus spike proteins. Annu Rev Virol.

[4] Shang, J., et al., Structure of 2019-nCoV chimeric receptor-binding domain complexed with its receptor human ACE2. Worldw. Protein Data Bank (2020).

[5] Shang J (2020). Cell entry mechanisms of SARS-CoV-2. Proc Natl Acad Sci.

[6] Li F (2015). Receptor recognition mechanisms of coronaviruses: a decade of structural studies. J Virol.

[7] Chen Y, Liu Q, Guo D (2020). Emerging coronaviruses: genome structure, replication, and pathogenesis. J Med Virol.

[8] Bahramali E (2016). Association of ACE gene D polymorphism with left ventricular hypertrophy in patients with diastolic heart failure: a case–control study. BMJ Open.

[9] Sekuri C (2005). Renin-angiotensin system gene polymorphisms and premature coronary heart disease. J Renin Angiotensin Aldosterone Syst.

[10] Firouzabadi N (2016). Genetic variants of angiotensin-converting enzyme are linked to autism: a case-control study. PLoS ONE.

[11] Vaduganathan M (2020). Renin–angiotensin–aldosterone system inhibitors in patients with Covid-19. N Engl J Med.

[12] Ferrario CM (1990). The renin-angiotensin system: importance in physiology and pathology. J Cardiovasc Pharmacol.

[13] Li X (2020). Impact of cardiovascular disease and cardiac injury on in-hospital mortality in patients with COVID-19: a systematic review and meta-analysis. Heart.

[14] Sabatino J (2020). Impact of cardiovascular risk profile on COVID-19 outcome. A meta-analysis. PLoS ONE.

[15] Shamshirian, A., et al., Cardiovascular diseases and COVID-19 mortality and intensive care unit admission: A systematic review and meta-analysis. medRxiv, 2020.

[16] Shenoy V (2010). The angiotensin-converting enzyme 2/angiogenesis-(1–7)/Mas axis confers cardiopulmonary protection against lung fibrosis and pulmonary hypertension. Am J Respir Crit Care Med.

[17] Wösten-van Asperen RM (2011). Acute respiratory distress syndrome leads to reduced ratio of ACE/ACE2 activities and is prevented by angiotensin-(1–7) or an angiotensin II receptor antagonist. J Pathol.

[18] Yan F (2020). Antihypertensive drugs are associated with reduced fatal outcomes and improved clinical characteristics in elderly COVID-19 patients. Cell Discov.

[19] Yang, G., et al., Angiotensin II receptor blockers and angiotensin-converting enzyme inhibitors usage is associated with improved inflammatory status and clinical outcomes in COVID-19 patients with hypertension. MedRxiv, 2020.

[20] Sun, M., et al., Inhibitors of RAS might be a good choice for the therapy of COVID-19 pneumonia. Zhonghua jie he he hu xi za zhi= Zhonghua jiehe he huxi zazhi= Chinese journal of tuberculosis and respiratory diseases, 2020. 43:E014-E014. 10.3760/cma.j.issn.1001-0939.2020.0014 32061198

[21] Wu J (2021). Advances in research on ACE2 as a receptor for 2019-nCoV. Cell Mol Life Sci.

[22] Letko M, Marzi A, Munster V (2020). Functional assessment of cell entry and receptor usage for SARS-CoV-2 and other lineage B betacoronaviruses. Nat Microbiol.

[23] Costa LB (2020). Insights on SARS-CoV-2 molecular interactions with the renin-angiotensin system. Front Cell Dev Biol.

[24] Hamming I (2004). Tissue distribution of ACE2 protein, the functional receptor for SARS coronavirus. A first step in understanding SARS pathogenesis. J Pathol J Pathol Soc Great Britain Ireland.

[25] Donoghue M (2000). A novel angiotensin-converting enzyme–related carboxypeptidase (ACE2) converts angiotensin I to angiotensin 1–9. Circ Res.

[26] Vickers C (2002). Hydrolysis of biological peptides by human angiotensin-converting enzyme-related carboxypeptidase. J Biol Chem.

[27] Chappel M, Ferrario C (2006). ACE and ACE2: their role to balance the expression of angiotensin II and angiotensin-(1–7). Kidney Int.

[28] Leisman DE, Deutschman CS, Legrand M (2020). Facing COVID-19 in the ICU: vascular dysfunction, thrombosis, and dysregulated inflammation. Intensive Care Med.

[29] Liu Z (2020). Composition and divergence of coronavirus spike proteins and host ACE2 receptors predict potential intermediate hosts of SARS-CoV-2. J Med Virol.

[30] Liu Y (2020). Clinical and biochemical indexes from 2019-nCoV infected patients linked to viral loads and lung injury. Sci China Life Sci.

[31] Nukiwa T (1982). Responses of serum and lung angiotensin-converting enzyme activities in the early phase of pulmonary damage induced by oleic acid in dogs. Am Rev Respir Dis.

[32] Dandona P (2007). Angiotensin II and inflammation: the effect of angiotensin-converting enzyme inhibition and angiotensin II receptor blockade. J Hum Hypertens.

[33] Liu F (2020). Prognostic value of interleukin-6, C-reactive protein, and procalcitonin in patients with COVID-19. J Clin Virol.

[34] Wiese O, Allwood B, Zemlin A (2020). COVID-19 and the renin-angiotensin system (RAS): A spark that sets the forest alight?. Med Hypotheses.

[35] Chaoxin J (2013). The influence of angiotensin-converting enzyme 2 gene polymorphisms on type 2 diabetes mellitus and coronary heart disease. Eur Rev Med Pharmacol Sci.

[36] Gemmati D, Tisato V (2020). Genetic hypothesis and pharmacogenetics side of renin-angiotensin-system in COVID-19. Genes.

[37] Alimoradi N, Firouzabadi N (2021). impact of genetics on predisposition and prognosis of COVID-19. Trends Pharmaceut Sci.

[38] Barash A (2020). The pursuit of COVID-19 biomarkers: putting the spotlight on ACE2 and TMPRSS2 regulatory sequences. Front Med.

[39] Srivastava A (2020). Genetic association of ACE2 rs2285666 polymorphism with COVID-19 spatial distribution in India. Front Genet.

[40] Çelik, S.K., et al., Polymorphisms of ACE (I/D) and ACE2 receptor gene (Rs2106809, Rs2285666) are not related to the clinical course of COVID-19; a case study. J Med Virol 2021. 10.1002/jmv.27160 PMC8426884 34170561

[41] Patel SK (2014). From gene to protein—experimental and clinical studies of ACE2 in blood pressure control and arterial hypertension. Front Physiol.

[42] Calcagnile M (2021). Molecular docking simulation reveals ACE2 polymorphisms that may increase the affinity of ACE2 with the SARS-CoV-2 Spike protein. Biochimie.

[43] Ashoor, D., et al., A computational approach to evaluate the combined effect of SARS-CoV-2 RBD mutations and ACE2 receptor genetic variants on infectivity: The COVID-19 host-pathogen nexus. bioRxiv, 2021: p. 2020.10. 23.352344. 10.3389/fcimb.2021.707194 PMC8381355 34434902

[44] Pouladi N, Abdolahi S (2021). Investigating the ACE2 polymorphisms in COVID-19 susceptibility: an in silico analysis. Mol Genet Genomic Med.

[45] Firouzabadi N (2011). Interaction of A-240T and A2350G related genotypes of angiotensin-converting enzyme (ACE) is associated with decreased serum ACE activity and blood pressure in a healthy Iranian population. Eur J Pharmacol.

[46] Firouzabadi N (2012). Association of angiotensin-converting enzyme (ACE) gene polymorphism with elevated serum ACE activity and major depression in an Iranian population. Psychiatry Res.

[47] Zhu X (2001). Linkage and association analysis of angiotensin I–converting enzyme (ACE)–gene polymorphisms with ACE concentration and blood pressure. Am J Human Genet.

[48] *COVID-19 Clinical management: living guidance*. 2021; Available from: https://www.who.int/publications/i/item/WHO-2019-nCoV-clinical-2021-1.

[49] Leger T (2020). Low-dose chest CT for diagnosing and assessing the extent of lung involvement of SARS-CoV-2 pneumonia using a semi quantitative score. PLoS ONE.

[50] MWer S, Dykes D, Polesky H (1988). A simple salting out procedure for extracting DNA from human nucleated cells. Nucleic Acids Res.

[51] Zhong J (2006). Association of angiotensin-converting enzyme 2 gene A/G polymorphism and elevated blood pressure in Chinese patients with metabolic syndrome. J Lab Clin Med.

[52] Iqbal MP (2004). Association study of the angiotensin-converting enzyme (ACE) gene G2350A dimorphism with myocardial infarction. Exp Mol Med.

[53] Firouzabadi N (2021). Impact of ACE 2 genetic variant on antidepressant efficacy of SSRIs. Acta Neuropsychiatrica.

[54] Firouzabadi N (2013). Gender specificity of a genetic variant of angiotensin-converting enzyme and risk of coronary artery disease. Mol Biol Rep.

[55] Kuba K (2005). A crucial role of angiotensin converting enzyme 2 (ACE2) in SARS coronavirus–induced lung injury. Nat Med.

[56] Wang, Q., et al., Structural and functional basis of SARS-CoV-2 entry by using human ACE2. Cell, 2020;181(4):894–904. e9. 10.1016/j.cell.2020.03.045 PMC7144619 32275855

[57] Santos RAS (2017). The ACE2/angiotensin-(1–7)/MAS axis of the renin-angiotensin system: focus on angiotensin-(1–7). Physiol Rev.

[58] Xu H (2020). High expression of ACE2 receptor of 2019-nCoV on the epithelial cells of oral mucosa. Int J Oral Sci.

[59] Rice GI (2004). Evaluation of angiotensin-converting enzyme (ACE), its homologue ACE2 and neprilysin in angiotensin peptide metabolism. Biochem J.

[60] Li Y (2016). Angiotensin-converting enzyme 2 prevents lipopolysaccharide-induced rat acute lung injury via suppressing the ERK1/2 and NF-κB signaling pathways. Sci Rep.

[61] Magalhaes GS (2018). Angiotensin-(1–7) promotes resolution of eosinophilic inflammation in an experimental model of asthma. Front Immunol.

[62] Wang D (2019). Renin-angiotensin-system, a potential pharmacological candidate, in acute respiratory distress syndrome during mechanical ventilation. Pulmonary Pharmacol Therapeut.

[63] He H (2015). Mesenchymal stem cells overexpressing angiotensin-converting enzyme 2 rescue lipopolysaccharide-induced lung injury. Cell Transplant.

[64] Bastos AC (2020). Oral formulation angiotensin-(1–7) therapy attenuates pulmonary and systemic damage in mice with emphysema induced by elastase. Immunobiology.

[65] Imai Y (2005). Angiotensin-converting enzyme 2 protects from severe acute lung failure. Nature.

[66] Magalhaes GS (2020). Activation of Ang-(1–7)/Mas receptor is a possible strategy to treat coronavirus (SARS-CoV-2) infection. Front Physiol.

[67] Chappell MC, Al Zayadneh EM (2017). Angiotensin-(1–7) and the regulation of anti-fibrotic signaling pathways. J Cell Signal.

[68] Gironacci MM (2015). Angiotensin-(1–7): beyond its central effects on blood pressure. Ther Adv Cardiovasc Dis.

[69] Wysocki J (2006). ACE and ACE2 activity in diabetic mice. Diabetes.

[70] Wu YH (2017). The ACE 2 G8790A polymorphism: involvement in type 2 diabetes mellitus combined with cerebral stroke. J Clin Lab Anal.

[71] Wang Z (2005). Immune responses with DNA vaccines encoded different gene fragments of severe acute respiratory syndrome coronavirus in BALB/c mice. Biochem Biophys Res Commun.

[72] Möhlendick B (2021). ACE2 polymorphism and susceptibility for SARS-CoV-2 infection and severity of COVID-19. Pharmacogenet Genom..

[73] Karakaş Çelik S (2021). Polymorphisms of ACE (I/D) and ACE2 receptor gene (Rs2106809, Rs2285666) are not related to the clinical course of COVID-19: a case study. J Med Virol.

[74] Gómez J (2020). Angiotensin-converting enzymes (ACE, ACE2) gene variants and COVID-19 outcome. Gene.

[75] Verdecchia P (2020). The pivotal link between ACE2 deficiency and SARS-CoV-2 infection. Eur J Intern Med.

[76] Mehta P (2020). COVID-19: consider cytokine storm syndromes and immunosuppression. Lancet.

[77] Akhmerov A, Marbán E (2020). COVID-19 and the heart. Circ Res.

[78] Hoffmann, M., et al., SARS-CoV-2 cell entry depends on ACE2 and TMPRSS2 and is blocked by a clinically proven protease inhibitor. Cell 2020;181(2):271–280.e8. 10.1016/j.cell.2020.02.052 PMC7102627 32142651

[79] Walls, A.C., et al., Structure, function, and antigenicity of the SARS-CoV-2 spike glycoprotein. Cell, 2020;181(2): 281–292.e6. 10.1016/j.cell.2020.02.058 PMC7102599 32155444

[80] Mahmood MS (2003). Association of the angiotensin-converting enzyme (ACE) gene G2350A dimorphism with essential hypertension. J Hum Hypertens.

[81] Schüler R (2017). High-saturated-fat diet increases circulating angiotensin-converting enzyme, which is enhanced by the rs4343 polymorphism defining persons at risk of nutrient-dependent increases of blood pressure. J Am Heart Assoc.

[82] Watanabe T, Barker TA, Berk BC (2005). Angiotensin II and the endothelium: diverse signals and effects. Hypertension.

[83] Senchenkova EY (2010). Angiotensin II–mediated microvascular thrombosis. Hypertension.

[84] Tay K-H, Lip GY (2008). What “drives” the link between the renin–angiotensin–aldosterone system and the prothrombotic state in hypertension?.

[85] Shi W, Lv J, Lin L (2020). Coagulopathy in COVID-19: Focus on vascular thrombotic events. J Mol Cell Cardiol.

[86] Biswas S (2021). Blood clots in COVID-19 patients: simplifying the curious mystery. Med Hypotheses.

[87] Ferreira AJ (2012). New cardiovascular and pulmonary therapeutic strategies based on the Angiotensin-converting enzyme 2/angiotensin-(1–7)/mas receptor axis. Int J Hypertens.

[88] Tan WSD (2018). Targeting the renin–angiotensin system as novel therapeutic strategy for pulmonary diseases. Curr Opin Pharmacol.

[89] Srivastava P (2019). Imbalance between Angiotensin II-Angiotensin (1–7) system is associated with vascular endothelial dysfunction and inflammation in type 2 diabetes with newly diagnosed hypertension. Diabetes Metab Syndr.

[90] Cook JR, Ausiello J (2021). Functional ACE2 deficiency leading to angiotensin imbalance in the pathophysiology of COVID-19. Rev Endocrine Metabolic Disord.

[91] Lanza K (2020). Covid-19: the renin–angiotensin system imbalance hypothesis. Clin Sci.

[92] Killerby ME (2020). Characteristics associated with hospitalization among patients with COVID-19—Metropolitan Atlanta, Georgia, March–April 2020. Morb Mortal Wkly Rep.

[93] Klein SL (2020). Biological sex impacts COVID-19 outcomes. PLoS Pathog.

